# The effects of low-fat dairy products fortified with 1500 IU vitamin D_3_ on serum liver function biomarkers in adults with abdominal obesity: a randomized controlled trial

**DOI:** 10.1186/s41043-023-00401-6

**Published:** 2023-09-25

**Authors:** Payam Sharifan, Susan Darroudi, Mahdi Rafiee, Kiarash Roustai Geraylow, Romina Hemmati, Mohammad Rashidmayvan, Mohamad Safarian, Saeid Eslami, Hassan Vatanparast, Reza Zare-Feizabadi, Maryam Mohammadi-Bjgiran, Hamideh Ghazizadeh, Zahra Khorasanchi, Mohammad Bagherniya, Gordon Ferns, Mitra Rezaie, Majid Ghayour-Mobarhan

**Affiliations:** 1 Department of Nutrition, School of Medicine, Mashhad University of Medical Sciences, Mashhad, Iran; 2 Student Research Committee, Faculty of Medicine, Mashhad University of Medical Sciences, Azadi Square, Mashhad, Iran; 3 International UNESCO Center for Health-Related Basic Sciences and Human Nutrition, Mashhad University of Medical Sciences, Mashhad, Iran; 4 Department of Medical Informatics, School of Medicine, Mashhad University of Medical Sciences, Mashhad, Iran; 5 College of Pharmacy and Nutrition, School of Public Health, University of Saskatchewan, Saskatoon, Canada; 6 Department of Community Nutrition, School of Nutrition and Food Science, Food Security Research Center, Isfahan University of Medical Sciences, Isfahan, Iran; 7 Division of Medical Education, Brighton and Sussex Medical School, Room 346, Mayfield House, Falmer, Brighton, BN1 9PH SSX UK

**Keywords:** Vitamin D, Abdominal obesity, Liver function test, Randomized controlled trial, RCT

## Abstract

**Introduction:**

Vitamin D deficiency has been reported to affect liver function biomarkers. This study was aimed to investigate the effect of consuming vitamin D fortified low-fat dairy products on liver function tests in adults with abdominal obesity.

**Methods:**

This total blinded randomized controlled trial was undertaken on otherwise healthy abdominally obese adults living in Mashhad, Iran. Milk and yogurt were fortified with 1500 IU vitamin D_3_ nano-capsules. Participants were randomized to receive fortified milk (*n* = 73), plain milk (*n* = 73), fortified yogurt (*n* = 69), and plain yogurt (*n* = 74) for 10 weeks. Blood samples were taken at baseline and at the end of the study to assess serum levels of vitamin D, alanine aminotransferase, aspartate aminotransferase, alkaline phosphatase (ALP), and Gamma glutamyl transferase.

**Results:**

A total of 289 participants completed the study (54% female). The groups were homogenous in terms of age, sex, weight, energy intake, and physical activity level (*p*-value > 0.05)**.** After the trial, vitamin D serum levels were significantly increased in both groups receiving fortified products (both *p* < 0.001). There was a significant time*group effect only in serum ALP (*p* < 0.001).

**Conclusion:**

Consumption of dairy products fortified by 1500 IU vitamin D_3_ might have detrimental effects on serum levels of some liver enzymes in individuals with abdominal obesity. Further studies needed to determine these effects and underlying mechanisms.

*Trial registration*: IRCT20101130005280N27.

## Introduction

Vitamin D is a fat-soluble vitamin that is derived from the diet and ultraviolet light skin exposure. Vitamin D is converted to its active forms by hydroxylase enzymes in the liver and kidneys [1]. Although vitamin D is known primarily for its role in mineral and skeletal homeostasis, its receptors are found in other tissues. Other effects of vitamin D include regulating cell differentiation, proliferation, apoptosis, and hormone secretion, as well as modulating the immune system. Also, it has a role in the pathogenesis of diabetes and cancer [2–5]. Vitamin D deficiency is a common condition worldwide. The prevalence of vitamin D deficiency among adults is between 5 and 30% globally, and 62% in Iran [6, 7]. The United States of America (USA) Endocrine Society recommends a constant daily intake of at least 1500–2000 IU to maintain serum 25(OH)D levels above 75 nmol/L to provide its potential non-musculoskeletal health benefits [8].

It should be noted that vitamin D deficiency is widely seen in patients with chronic liver disease, and it can be a risk factor for increased liver transaminases [9–11]. Previous studies have determined a significant role for vitamin D supplementation on liver enzymes or fibrosis level both among patients with fatty liver disease [12–14] and healthy individuals [15]. However, the studies differed in the magnitude of the effects of vitamin D supplementation on liver. The reported effects ranged from decrease in all or some of the liver enzymes, including alanine transaminase (ALT), to reduced liver fibrosis [12–15]. The possible mechanisms for these effects include the effects of vitamin D on weight reduction, oxidative stress, and hepatic regeneration [16, 17].

These findings and recommendations lead to the consumption of vitamin D fortified foods with suitable formulations in several countries to prevent vitamin D deficiency and its consequences [18, 19]. Among the food groups, dairy products are considered good choices for vitamin D fortification due to their calcium content [20]. The choice of dairy products for vitamin D fortification was based on the primary objective of improving bone density caused by vitamin D deficiency [18, 19]. In Iran, milk and yogurt, fermented form of dairy products, are considered as the best choices for vitamin D fortification [21]. However, the superiority of these product forms over each other has not yet been evaluated. Moreover, the bioavailability of vitamin D from dairy products is questioned [22]. A solution to overcome decreased bioavailability of vitamin D is to use nano-emulsion delivery systems [23].

Besides vitamin D deficiency, obesity, especially abdominal obesity, which is becoming more prevalent due to sedentary lifestyle, is a crucial risk factor for liver diseases, particularly non-alcoholic fatty liver disease (NAFLD), which is demonstrated by increased liver function tests [24, 25]. It should be taken into account that using fortified food staples especially dairy products (with high branched-chain amino acids) could be more beneficial for improvement of liver function biomarkers. Therefore, it can be hypothesized that vitamin D supplementation through food fortification in abdominally obese individuals can prevent the initiation (primary prevention) or progression (secondary prevention) of NAFLD. To the best of our knowledge, the effects of vitamin D supplementation with different fortified dairy products on liver function tests have not yet been studied in healthy but abdominally obese individuals. The objective of the present study was to investigate the effect of two low-fat vitamin D fortified dairy products with 1500 IU nano-encapsulated vitamin D_3_ on serum liver enzymes in abdominally obese participants.

## Materials and methods

### Study design

**Fig. 1:**
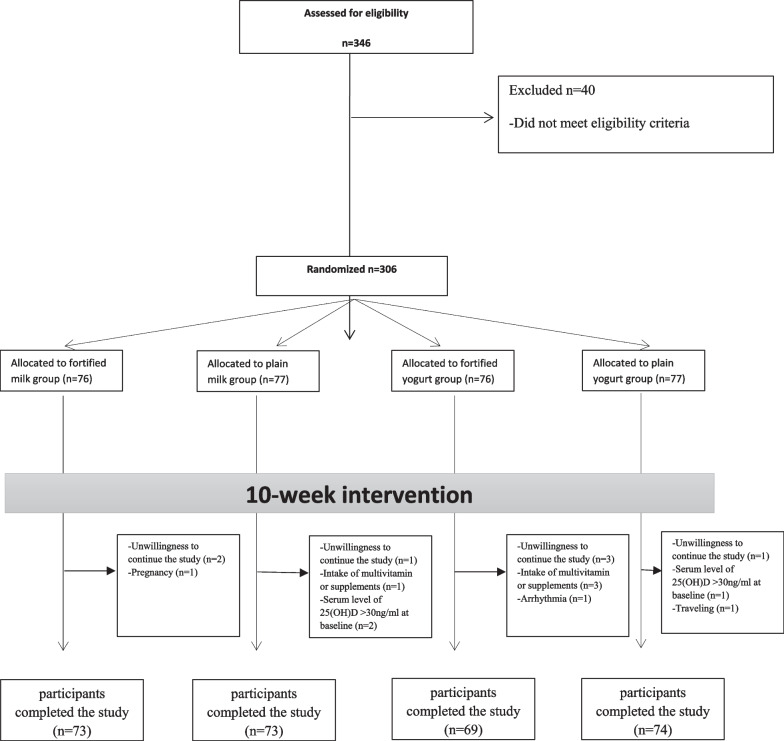
Flow chart of the study

This report is a part of the pilot study entitled: *Survey of Ultraviolent Intake by Nutritional Approach* (SUVINA) trial. A sub-analysis report of SUVINA was published previously [26]. We conducted a parallel quadruple (total) blinded randomized controlled trial (RCT) between January 2019 and March 2019 (in winter). The study duration was 10 weeks (Fig. 1). This study was conducted on 289 staff and students working at the Mashhad University of Medical Sciences, Mashhad, Iran. Mashhad is located in the North-East of Iran at 36.26° North latitude (temperate zone), in which the ultraviolet intake is estimated at minimum levels in winter months.

### Sample size

The sample size was calculated based on the power of 80%, effect size of 0.5. The sample size was calculated as 255 participants. After considering 10% dropout, the final sample size was determined as at least 280 participants (70 participants in each group) [27].

### Ethics

This trial received ethical approval from the Research Ethics Committee of the Iran National Institute for Medical Research Development (protocol ID: IR.NIMAD.REC.1396.027). The study was also registered at the Iran Registry of Clinical Trials at http://www.IRCT.ir (registration ID: IRCT20101130005280N27). Written informed consent was obtained from all participants prior to baseline evaluations.

### Inclusion and exclusion criteria

Participants were recruited among Mashhad University of Medical Sciences (MUMS) staff, students, and their relatives who consented to take part in our study. Participants were middle-aged adults (30–50 years) with abdominal obesity (waist circumference of 80 cm or higher for females and 94 cm or higher for males) based on proposed cutoff points of the International diabetes federation [28].

Criteria for excluding the participants were as follows: a history of lactase deficiency or any sensitivity to dairy products, hepatic or renal dysfunctions, smoking, alcohol consumption, pregnancy, lactation, using medications that interact with vitamin D (such as anticonvulsants or corticosteroids), using supplements containing vitamin D, or medications for mood, or sleep disorders; a current weight > 150 kg, weight changes > 5 kg in the previous year, planning to reduce weight, performing high-intensity physical activity, or having a special diet such as vegetarianism.

According to the socio-cultural characteristics of the Iranian population (kind of clothing, particularly for women) [29], and the study population who were staff and students of the medical sciences university (with less outdoor activities during the day), sun exposure was potentially lower than usual. Therefore, the confounding effect of sun exposure during the trial was considered meager.

### Procedure

Recruitment occurred one month prior to the intervention (November 2018). The screening was conducted in three steps: (1) phone conversation, (2) visiting participants at the clinic and clarifying the study protocol, (3) visiting participants to obtain informed consent a few days after the initial visit. Then a general physician who was briefed about the study procedure obtained a medical history and performed physical examination, including height, body weight, and waist circumferences.

The outcome parameters were assessed at baseline and the end of the study. Three-day-food records (including two working days and one weekend or holyday) were taken at baseline and at weeks 5 and 10 to obtain energy and nutrient intakes. For this reason, all dietary data were converted into grams and were entered into the Nutritionist 4 software (First Databank, San Bruno, CA, United States) based on the United States Department of Agriculture (USDA) food composition table. The database was modified to include Iranian foods. Physical Activity Level (PAL) was assessed by Beck physical activity questionnaire [30, 31]. This 16-items questionnaire is categorized into three indices; work index, sports index, and free time index. The severity of physical activity was measured by the summation of these three indices. This questionnaire was validated on Iranian population by Etemad et al. [31] (Cronbach’s alpha = 0.79). All participants were asked not to change their regular diet and physical activity during the trial.

### Anthropometric assessments

Height was assessed using a wall stadiometer at baseline. A digital bio impedance analyzer (Tanita BC 418; Japan) was applied to evaluate weight, fat mass percentage (FM%), and fat-free mass percentage (FFM%) at baseline and after ten weeks of intervention. Body mass index was calculated using the following formula:

$${\text{Body}}\;{\text{mass}}\;{\text{index}} = {{{\text{weight}}\left( {{\text{kg}}} \right)} \mathord{\left/ {\vphantom {{{\text{weight}}\left( {{\text{kg}}} \right)} {{\text{height}}^{2} \left( {{\text{m}}^{2} } \right)}}} \right. \kern-0pt} {{\text{height}}^{2} \left( {{\text{m}}^{2} } \right)}}$$

Waist circumference was measured twice by a single expert staff using a flexible non-elastic tape at the midpoint between iliac crest edge and bottommost rib at the end of a normal exhalation [32].

### Blood sample measurements

After a 12-h over-night fasting, 20 ml of venous blood was taken from a brachial vein of eligible participants to evaluate serum levels of vitamin D, ALT, aspartate aminotransferase (AST), alkaline phosphatase (ALP), and Gamma glutamyl transferase (GGT). All blood samples were collected in EDTA anticoagulant tubes in the morning. Samples were then centrifuged at 5000 g for 15 min at 4 °C to separate the serum and its aliquots. Samples were stored immediately at − 80 °C.

Serum 25(OH)D concentrations were measured by commercial ELISA kits (Pishgaman sanjesh- Iran), using an Awareness/Stat Fax 2100 analyzer [33]. AST, ALT, and ALP were assessed using the Pars Azmun kits on a BT-3000 auto-analyzer (Biotechnical, Rome, Italy) [34].

### Randomization and blinding

Stratified block randomization was performed according to centers and sex, using a randomized block design with the ratio of 1:1:1:1. Participants were allocated to one of four groups as follows: fortified milk (FM), fortified yogurt (FY), plain milk (PM), and plain yogurt (PY). A staff member who was not involved in data collection, analysis, and reporting accomplished the random allocation using sequentially numbered, opaque, sealed envelopes.

Blinding was at four levels (total blinding): participants, investigators, outcome assessors, statistician and researcher responsible for randomizing subjects.

### Intervention

For 10 weeks, each participant was given a portion of dairy product according to their groups (200 ml milk or 150 ml yogurt in a disposable plastic container). Participants were instructed to consume the products daily, as far as possible in front of the researcher in morning snack time (around 9–11 AM). For better blinding, we used two different product numbers to divide 1500 IU vitamin D fortified products from placebos.

Dairy products were distributed daily with a specific code labeled on each glass for better control of receipt by the subjects. Products were delivered on the previous day for weekends and holidays to ensure daily consumption. Also participants were asked to return empty glasses on the day after the weekend.

### Production of nano-encapsulated vitamin D_3_

The nano-encapsulated formulation was produced by components included: Precirol (glyceryl palmitostearate) as the solid lipid, poloxamer 188 as the non-ionic surfactant, oleic acid as the liquid lipid, vitamin D as the bioactive fatty core, and deionized water. The first three components mentioned above are inactive ingredients approved by the Food and Drug Administration. All components were mixed by homogenization with high tensile stress and ultrasound. The prepared Nano-Lipid Carriers (NLCs) had an average size of 126.3 ± 10.81 nm and a negative charge of − 17.2 ± 1.65 mv. Also, a polydispersity index (PDI) value of 0.107 ± 0.002 to 0.209 ± 0.017 was detected, indicating a narrow distribution of particle size for NLCs.

Production and the fortification process of low-fat dairy products were carried out at the Salamat pilot dairy product factory under the supervision of the Faculty of Food Sciences and Technology, Ferdowsi University of Mashhad, Iran. Each 100 g of milk and yogurt in this study includes 56 kcal, 7 gr protein, sugar-free, 3 g fat, and 0.04 g trans fatty acids. Delivery and consumption of products were done on production day or the next day after.

### Post-intervention assessments

Post-intervention assessments took place immediately after the end of the intervention. All anthropometric and blood parameters that have been measured at baseline, including weight, BMI, serum vitamin D, ALT, AST, ALP, GGT, were measured.

### Statistics analysis

The Kolmogorov–Smirnov test (KS test) was used to assess the normality of quantitative data. Quantitative variables were described as means ± standard deviation (SD), while the qualitative variables were expressed as percentage and frequency and were compared using the Chi-square test. The repeated measures analysis of covariance (ANCOVA) was used to assess the effect of group, time, and time*group in terms of the outcome variables with ALT, AST, and ALP as dependent factors, intervention groups as within and time as between-subject factors and fasting blood glucose, serum cholesterol, triglycerides, waist circumference, waist: hip ratio, and serum vitamin D at baseline as covariates.

In this study, only three participants had vitamin D levels higher than 30 ng/ml. After performing sensitivity analysis, excluding these three participants did not significantly affect the results of the statistical analyses. Therefore, in order to obtain a homogeneity in terms of serum vitamin D levels, these three participants were excluded from the study.

All statistical analyses were performed using the Statistical Package for the Social Sciences (SPSS) software version 16. A *p*-value of less than 0.05 was considered as statistically significant.

## Results

Among the original 306 eligible participants, 17 participants were excluded from analyses for the following reasons: refusal to continue the study (*n* = 7), serum vitamin D level > 30 ng/ml at baseline (*n* = 3), using supplements or multivitamins during the trial (*n* = 4), becoming pregnant (*n* = 1), developing cardiac arrhythmia (*n* = 1), and loss to follow up (*n* = 1). Finally, 289 participants finished the trial, including 73 participants in the fortified milk and plain milk groups, 69 participants in the fortified yogurt, and 74 participants in the plain yogurt group for 10 weeks.

The demographic data of the study participants are presented in Table 1. The groups were homogenous in terms of age, sex, weight, energy intake, and physical activity level (*p*-value > 0.05). The mean age of our study participants in the fortified group and the plain group was 41.9 ± 7.77 and 41.75 ± 7.88 years and the mean BMI was 23.37 ± 3.37 and 23.19 ± 3.22 kg/m^2^.

**Table 1:** Demographic data of the study participants

	Milk		*P*-value	Yogurt		*P*-value
	Fortified	Plain		Fortified	Plain	
Age (year)*	40.42 ± 8.03	40.26 ± 8.25	0.9	43.47 ± 7.21	43.19 ± 7.25	0.82
Male	34 (49.3%)	36 (52.2%)	0.43	30 (46.2%)	32 (45.1%)	0.51
Female	35 (50.7%)	33 (47.8%)		35 (53.8%)	39 (54.9%)	
Waist circumference (cm)*	94.36 ± 9.28	94.02 ± 9.79	0.83	94.46 ± 8.94	94.34 ± 8.47	0.93
BMI (kg/m^2^)*	22.95 ± 2.96	23.11 ± 3.19	0.77	23.8 ± 3.65	23.27 ± 3.27	0.37
Energy intake (kcal/day)	2132.25 ± 775.81	1994.03 ± 514.47	0.71	1895.44 ± 597.31	2090.97 ± 758	0.758
Physical activity level (PAL)	6.58 ± 1.49	6.56 ± 1.42	0.95	6.41 ± 1.79	6.17 ± 1.39	0.40

*Mean and standard deviation were presented, and the independent t test was used for the comparison. Frequency and percentage were presented and the Chi-square test was used for the rest of the variables

The repeated measures ANCOVA revealed a significant group effect for GGT (*p* = 0.002) and vitamin D (*p* = 0.016). A significant time effect was found for ALT (*p* = 0.006), AST (*p* = 0.012) and ALP (*p* = 0.019) and a significant time*group effect was found for ALP (*p* < 0.001) (Tables 1 and 2).

**Table 2:** Comparison of liver function tests within and between time points and groups

Group	Intervention	Time	ALT Mean ± SD	AST Mean ± SD	ALP Mean ± SD	GGT Mean ± SD	Vitamin D
Milk	Fortified	Baseline	22.20 ± 15.21	22.96 ± 7.34	227.46 ± 61.74^a^	18.00 ± 9.25	13.87 ± 5.22
		After	17.74 ± 11.36	19.84 ± 11.79	189.99 ± 49.48	16.04 ± 7.56	19.13 ± 5.80
		Mean difference ± SE	− 4.36 ± 1.32	− 3.01 ± 1.37	− 37.10 ± 3.33	− 1.931.38	5.24 ± 0.46
		*p* (within)	0.003*	0.047*	< 0.001*	0.261	< 0.001*
	Plain	Baseline	23.58 ± 15.75	22.96 ± 7.34	213.87 ± 51.43	23.24 ± 14.25	14.26 ± 5.04^b^
		After	20.26 ± 12.04	21.26 ± 16.62	186.52 ± 42.00	21.98 ± 16.74	14.16 ± 5.69^c^
		Mean difference ± SE	− 3.18 ± 1.33	− 1.59 ± 1.38	− 26.77 ± 3.37	− 1.04 ± 1.39	− 0.09 ± 0.46
		*p* (within)	0.021*	0.243	< 0.001*	0.363	< 0.001*
Yogurt	Fortified	Baseline	22.03 ± 15.85	22.35 ± 11.29	204.38 ± 46.31^a^	24.06 ± 18.25	14.20 ± 5.05^b^
		After	22.40 ± 15.56	20.80 ± 7.32	193.00 ± 46.21	25.36 ± 24.08	21.11 ± 5.69^c^
		Mean difference ± SE	0.09 ± 1.38	− 1.73 ± 1.43	− 11.93 ± 3.47	1.10 ± 1.44	6.92 ± 0.48
		*p* (within)	0.862	0.366	0.058	0.351	< 0.001*
	Plain	Baseline	22.03 ± 18.11	22.58 ± 8.42	209.70 ± 49.27	20.63 ± 11.98	15.42 ± 5.74
		After	19.62 ± 12.28	20.69 ± 7.24	189.20 ± 46.97	19.13 ± 11.67	14.47 ± 3.97
		Mean difference ± SE	− 1.26 ± 1.31	− 1.39 ± 1.36	− 20.95 ± 3.31	− 1.57 ± 1.37	− 0.95 ± 0.45
		*p* (within)	0.334	0.291	< 0.001*	0.207	< 0.001*

*ALT* alanine aminotransferase, *AST* aspartate aminotransferase, *ALP* alkaline phosphatase, *GGT* gamma glutamyl transferase, *Vit D* vitamin D

Repeated measures analysis of covariance was conducted controlling for fasting blood glucose, serum cholesterol, triglycerides, waist circumference, waist: hip ratio

*Significant difference

^a^*p* = 0.046

^b^*p* = 0.010

^c^*p* = 0.010

Comparison of the serum vitamin D concentration and liver function biomarkers are presented in Table 3. The intervention resulted in a significant decrease in serum vitamin D in fortified milk and yogurt groups (*p* < 0.001 each), while serum vitamin D decreased significantly after intervention duration in plain milk and yogurt groups (*p* < 0.001 each). There was a significant difference in serum vitamin D at baseline and the end of intervention between plain milk and fortified yogurt (*p* = 0.010 each) (Table 3). Intervention resulted in a significant reduction in serum ALT in fortified and plain milk groups (*p* = 0.003 and *p* = 0.021, respectively) (Table 3). The intervention resulted in a significant decrease in serum AST only in the fortified milk group (*p* = 0.047). Intervention resulted in a significant decrease in serum ALP in fortified and plain milk and plain yogurt groups (*p* < 0.001 each). There was a significant difference in serum ALP between baseline values of fortified milk and fortified yogurt (*p* = 0.046) (Table 3).

**Table 3:** Results of repeated measures analysis of covariance for liver function tests

Variable	Group effect				Time effect				Time*group effect			
	*F*	*p*	Partial eta squared	Power	*F*	*p*	Partial eta squared	Power	*F*	*p*	Partial eta squared	Power
ALT	0.807	0.308	0.004	0.174	7.783	0.006*	0.029	0.794	2.154	0.094	0.024	0.545
AST	0.198	0.897	0.002	0.063	6.437	0.012*	0.024	0.715	0.211	0.889	0.002	0.089
ALP	0.537	0.657	0.006	0.160	5.596	0.019*	0.021	0.654	10.987	< 0.001*	0.111	0.999
GGT	5.204	0.002*	0.054	0.914	0.410	0.523	0.002	0.098	0.990	0.398	0.011	0.268
Vit D	3.520	0.016*	0.038	0.779	< 0.001	0.999	< 0.001	0.050	< 0.001	0.999	< 0.001	0.050

*ALT* alanine aminotransferase, *AST* aspartate aminotransferase, *ALP* alkaline phosphatase, *GGT* gamma glutamyl transferase, *Vit D* vitamin D

Repeated measures analysis of covariance was conducted controlling for fasting blood glucose, serum cholesterol, triglycerides, waist circumference, waist: hip ratio

The degree of freedom for group effects, time effects and time*group effects was 3, 1, and 3, respectively, for all variables

*Significant effect

**Fig. 2:**
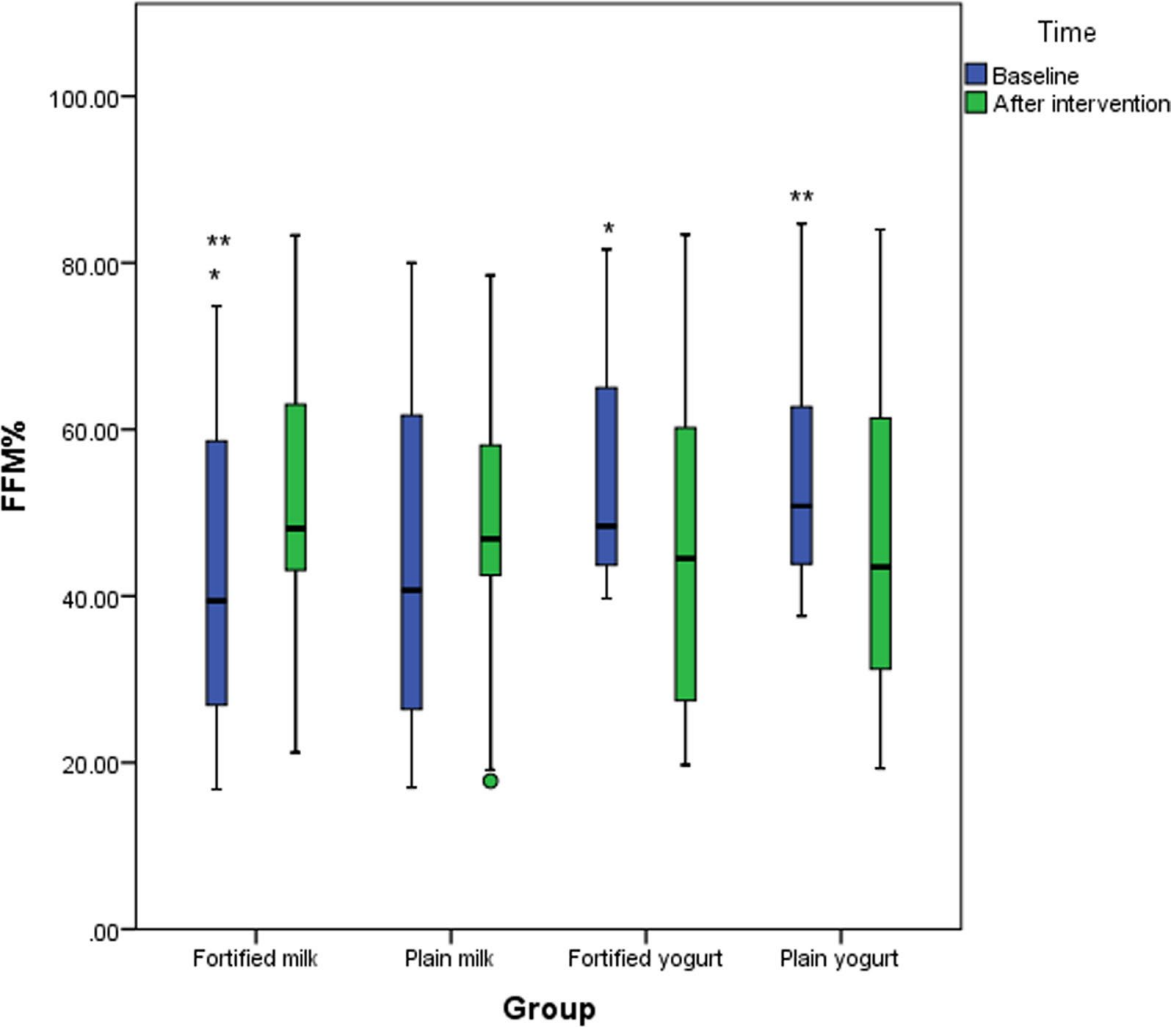
Changes in fat mass percentage (FM%) at study time points among study groups

There was a significant difference in terms of FFM% between fortified milk and plain yogurt and fortified yogurt at baseline (both *p* < 0.001) (Fig. 2). No significant difference in FM% was observed between groups at time points.

## Discussion

This study was the first study that assessed the effects of low-fat dairy products fortified with nano-encapsulated vitamin D on liver function parameters in adults with abdominal obesity. After ten weeks of intervention, treatment with vitamin D fortified dairy products was associated with a significant, increase in serum vitamin D concentrations, although serum 25(OH)D levels did not reach a sufficient serum concentration (> 30 ng/ml). A significant reduction from baseline levels was observed in serum ALP in both the fortified dairy groups compared to unfortified dairy products. This finding was consistent with previous studies [35, 36]. ALP is an important serum biomarker. Elevation of serum ALP levels is associated with several liver diseases and can also demonstrate bile duct obstruction [37]. This inverse association of vitamin D intake and ALP serum levels in our study may show the potential role of vitamin D in preventing liver tissue injuries.

Nam et al. reported that the activity of aminotransferases was positively correlated with serum levels of vitamin D, and it has been shown that vitamin D deficiency is an independent risk factor for elevated aminotransferases in obese individuals [38, 39]. Furthermore, Dabbaghmanesh et al. [40] showed that serum ALP and GGT levels were significantly reduced in the patients with FAFLD who received vitamin D after the intervention. In their study, participants were given 50,000 IU vitamin D3 (cholecalciferol) capsule per week and 0.25 mg calcitriol (1,25 dihydroxycholecalciferol) pearl per day for 3 months [40]. These findings were similar to the findings of our study except for a significant reduction in serum GGT. This difference may be due to differences in the dosage and duration of administered vitamin D as well as the study participants. Furthermore, unlike our study, in the study by Dabbaghmanesh et al. vitamin D supplementation optimized serum 25(OH)D_3_. Therefore, it may be hypothesized that vitamin D supplementation at higher doses of vitamin D for a longer duration might be needed to affect serum GGT. It may also be hypothesized that ALP is a more sensitive marker that can show vitamin D related improvements in liver function even with suboptimal elevations in serum 25(OH)D_3_ concentration.

In our study, the ALT and AST were reduced in all groups except for fortified yogurt. This finding was in contrast with the findings of the mentioned studies. In contrast, few studies reported no association between serum levels of vitamin D and AST and ALT in patients with NAFLD who were supplemented with oral capsule consisting of 50,000 IU vitamin D3 [41, 42]. Furthermore, a meta-analysis stated that vitamin D supplementation has no effect on AST or ALT [43, 44]. The reason for this difference might be due to the difference in the dose of vitamin D supplementation as well as the study participants, who were NAFLD patients, while the participants in our study were obese adults with no documented diagnosis of NAFLD. Although our study participants had almost normal levels of ALT and AST, we could not disregard that some of them had NAFLD or even non-alcoholic steatohepatitis (NASH), because liver enzymes were demonstrated to be neither sensitive nor specific in the diagnosis of chronic liver disease [45].

It might also be hypothesize that the improvement in ALT and AST in our study might be due to the effect of consumption of dairy products, not vitamin D supplementation. Animal studies reported that branched-chain amino acids (BCAAs) from casein in milk reduced hepatic apoptosis in Sprague Dawley rats. Hepatocyte function was altered by BCAAs, which was observed by the improvement of hepatocyte aminotransferases activity [46]. A study on human subjects with metabolic syndrome showed that consumption of three servings of dairy products per day could improve liver enzymes and systemic inflammation [47]. In our study, although ALT and AST were significantly reduced in the plain yogurt group, no significant change in ALT and AST was found in the fortified yogurt group. Furthermore, no significant time*group effect was observed for ALT and AST in our study. This result indicates that the observed changes within groups over time were not significantly different. These findings may question the strength of the effect of dairy products consumption on liver transaminases, but there is a need for further studies to assess this effect, especially in patients with elevated liver transaminases.

The observed changes in liver function biomarkers may be caused via the VDR on the liver. VDR has been widely detected in the liver, and the performance pattern of this receptor is through connection to its own ligand and entering the nucleus from the cytoplasm [48]. It previously was demonstrated that human hepatocytes use VDR proteins for inhibition against cholestatic injury [49]. Also, hepatic stellate cells, sinusoidal endothelial cells, and Kupffer cells firmly express VDR [50]. This may support the observed effect of vitamin D supplementation on liver function in the non-diseased state. Previous studies suggested that weight loss is beneficial for the improvement of liver function biomarkers [51–53]. As the weight changes were not significant in our study, it could be an evidence for the independent effect of vitamin D on liver function biomarkers from mechanisms other than weight loss or adipocyte changes. It might also be hypothesized that the reduced serum ALP levels might be the result of improving gallbladder ejection fraction. It was previously shown that vitamin D supplementation resulted in 20% increase in gallbladder ejection fraction in patients with cholelithiasis [54, 55]. The mechanism for this effect was attributed to increased smooth muscle contractility due to the nuclear and non-nuclear VDRs as well as improving secondary hyperparathyroidism due to vitamin D deficiency [54]. These hypotheses may not be proved or rejected as none of our study did not assess gallbladder function nor did it include patients with gallstone related symptoms. As gallbladder function is related to liver function, this mechanism may serve as an indirect mechanism for the effects of vitamin D on liver function.

Milk has been known for its myotropic effects due to its various nutritional components; although, this effect is obtained by high amount of intake along with exercise [56]. Finally, a meta-analysis published by Abarghouei et al. [57] has demonstrated that an increase in dairy product consumption without energy restriction might not accelerate an increase in lean body mass in adults. Therefore, it is recommended that further studies assess the effect of different dairy products on FFM in individuals with metabolic syndrome.

The sample size and the design of this study (total blind RCT) can be considered as the strengths of this study. Furthermore, this study was the first study to investigate the efficacy of low-fat dairy products fortified with nano-encapsulated vitamin D on liver function and relative enzymes.

One of the limitation points of this study was not investigating the presence of fatty liver in participants. This factor could be effective in interpreting the results. However, this restriction did not significantly affect our results as evidenced by similar findings reported by previous studies. Study duration might be another limitation of our study. Since the data in this study were obtained from a larger study with a different objective, the duration of the study could not be altered. There is a need for further studies to assess the effects of vitamin D supplementation on liver function biomarkers for longer periods. Furthermore, this study only evaluated the final outcome of vitamin D fortification on liver enzymes in abdominally obese individuals. Therefore, there is a need for further studies to evaluate the possible mechanisms of this effect.

## Conclusion

The findings of this study indicated that both the fortified products improved serum vitamin D and that fortified milk might be a better choice in terms of improving liver enzymes in abdominally obese individuals.

## Data Availability

Data can be supplied on request to the authors.

## Abbreviations

AST: Aspartate aminotransferase
ALT: Alanine aminotransferase
ALP: Alkaline phosphatase
GGT: Gamma glutamyl transferase
BMI: Body mass index
VDR: Vitamin D receptor
